# Late Developing Supernumerary Premolars: Reports of Two Cases

**DOI:** 10.1155/2013/969238

**Published:** 2013-01-08

**Authors:** Soghra Yassaei, Mahdjoube Goldani Moghadam, S. M. Ali Tabatabaei

**Affiliations:** ^1^Department of Orthodontics, Faculty of Dentistry, Shahid Sadoughi University of Medical Sciences, Yazd 89195/165, Iran; ^2^Private Practice, Yazd 89195/165, Iran

## Abstract

This paper presents two cases of late developing supernumerary premolars. Case 1 is a female patient with nonsyndromic multiple supernumerary teeth located in the maxillary right premolar-molar region, maxillary left premolar region, and the mandibular right and left premolar regions. In this patient surgical removal of all supernumerary teeth was carried out to avoid complications during orthodontic treatment. Case 2 is a female aged 19 years in whom formation of a mandibular supernumerary premolar was observed which was not present at age 13. The patient was made aware of the supernumerary tooth presence, and periodic radiographic assessment was planned.

## 1. Introduction

Excess in the number of teeth which is often referred to as supernumerary is a developmental anomaly [1]. Etiologically, the occurrence of supernumerary teeth has not been yet completely determined. In attempts to explain the condition several theories have been proposed including the phylogenetic theory [2], the dichotomy theory (splitting of the tooth germ) [3, 4], the hyperactivity theory (hyperactive dental lamina) [1], and occurrence due to combined genetic and environmental effects [5]. Supernumerary teeth can occur anywhere in the dental arch, but they are mostly found in the maxilla [6]. The prevalence of supernumerary teeth varies between 0.1% and 3.8%, and they are more common in the permanent dentition [7]. Supernumerary teeth can be categorized in terms of chronology, topography, and morphology. Chronologically, they can develop before deciduous teeth (predeciduous), simultaneous to permanent teeth or after them (postpermanent dentition). Based on the morphology, supernumerary teeth can be defined as supplemental (eumorphic) or rudimentary (including conic shape, tuberculate, molariform, and odontome). Mesiodens, paramolar, distomolar, and parapremolar, are words to describe the topography (location) of the supernumeraries [7].

Supernumerary premolars represent between 8 and 9.1 percent of all supernumerary teeth [8, 9]. They are more likely to develop in the mandible than in the maxilla and often resemble the normal premolars in shape and size [10]. 

The majority of supernumeraries in the permanent dentition develop later than normal teeth of that region and are supposed to form postpermanent dentition developing from hyperactivity of dental lamina [11]. The presence of supernumeraries is associated with complications including delayed eruption of the permanent teeth, displacement of them, cyst formation, and resorption of neighboring structures such as root resorption [6]. They can also interfere in orthodontic space closure and implant placement [7]. When supernumeraries are diagnosed, a decision whether to monitor or remove them is needed to be made. In special situations removal of supernumeraries is preferred such as when the teeth need to be aligned orthodontically. Occasionally, supernumerary teeth are detected incidentally during radiographic examination. Regarding surgical risks, when complications associated with supernumeraries seem unlikely, periodic radiographic assessment would be recommended. Radiographic assessment is of great importance in diagnosis and management of supernumeraries. Advances in radiographic techniques and introduction of three-dimensional computed tomography (3D CT) and cone beam computed tomography (CBCT) have allowed undistorted view and better evaluation of supernumeraries especially in cases of multiple supernumeraries [12].

There have been reports of late developing supernumeraries in the literature [13–16]. Two further cases of late developing supernumerary premolars and two different approaches for their management are described in this paper.

## 2. Case Presentation

### 2.1. Case  1

**Figure 1:**
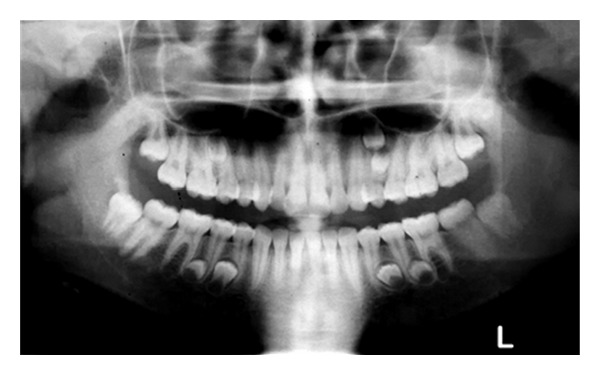
Panoramic radiograph of the patient showing the presence of seven supernumerary premolars.

The patient was a 15-year-old Iranian girl who was referred to the orthodontic clinic for treatment of Class II malocclusion. Family medical history was noncontributory and extraoral examination did not show any abnormality. In the panoramic view, the presence of seven supernumerary teeth resembling premolars which were located in the maxillary right premolar-molar region, maxillary left premolar region, and the mandibular right and left premolar regions was detected (Figure 1).

**Figure 2:**
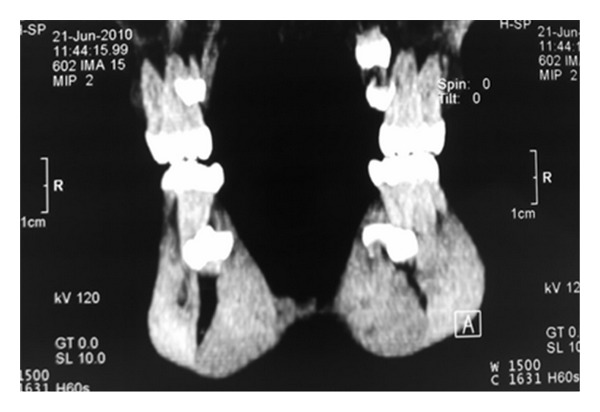
CT scan to evaluate the position of supernumeraries.

**Figure 3:**
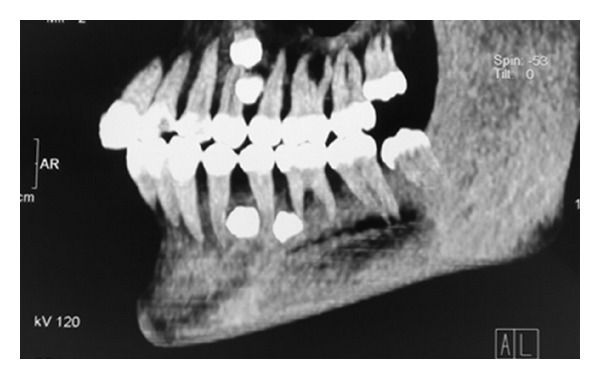
CT scan view of the left side to locate exact position of four supernumeraries.

**Figure 4:**
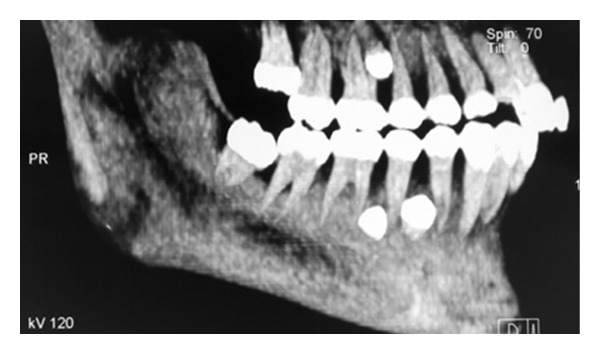
CT scan view of the right side showing three supernumeraries.

A general physician was consulted who confirmed there was no associated syndrome. The situation was explained to the patient and her parents. Further CT scans were taken to evaluate the position of the supernumeraries (Figures 2, 3, and 4).

The orthodontic treatment plan included extraction of upper second premolars, banding of first molars and bracket bonding on the remaining teeth, aligning, leveling, canine retraction, and anterior retraction. In order to prevent root resorption during initial phase of aligning and leveling and also to avoid incomplete space closure during canine retraction, a decision was made to surgically remove all supernumerary teeth.

### 2.2. Case  2

**Figure 5:**
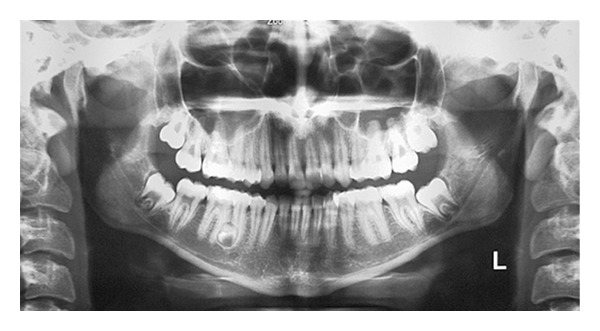
Panoramic radiograph taken at 19 years of age showing development of a supernumerary premolar.

**Figure 6:**
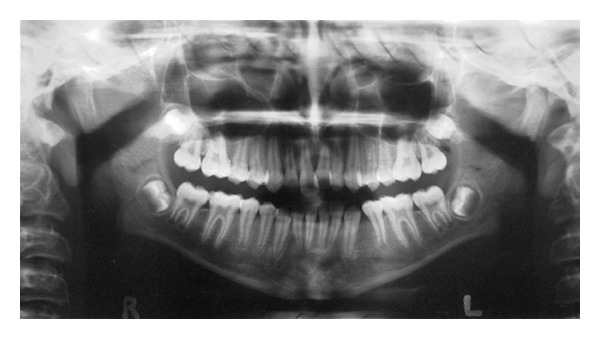
Panoramic radiograph of the same patient as in Figure 5 at 13 years of age showing no sign of the supernumerary tooth.

A 19-year-old Iranian female visited a general dental practitioner to asses her third molars. A panoramic radiograph was taken to evaluate the third molar teeth which revealed the presence of a supernumerary premolar in early stage of crown formation between the roots of mandibular right second premolar and first molar (Figure 5). The patient had been treated for her mild crowding at the age of 13 with nonextraction approach (the mandibular left first premolar had been extracted before referring the patient for orthodontic treatment), and her preorthodontic treatment panoramic radiograph was available. The old panoramic radiograph showed normal dentition, and there was no sign of supernumerary tooth (Figure 6). In this case the patient became aware of the condition and periodic monitoring of the supernumerary tooth and the third molar teeth by radiographic examination was planned.

## 3. Discussion

Supernumerary teeth may appear as a single tooth or multiple teeth. Multiple supernumeraries are not common finding, and occurrence of five or more supernumeraries has been estimated less than 1 percent [17]. Multiple supernumeraries are more likely to occur as part of a syndrome such as Cleidocranial dysplasia, Gardner's syndrome, orodigitofacial dystosis, Down's syndrome, Crouzon's disease, and Hallermann Streiff syndrome but can also occur without such an association [7, 17]. 

As stated earlier about 8–9.1 percent of all supernumerary teeth are supernumerary premolars [8, 9]. Unlike other supernumeraries, supernumerary premolars are more prevalent in the mandible and usually resemble normal premolars in shape and size [6]. These supernumeraries have tendency to begin their development later than the normal for teeth of that area [7]. It has been shown that calcification of premolars initiates between 1.5 and 2.5 years of age while it may not be evident until 3-4 years of age in radiographic views [18]. It has been reported that supernumerary premolars commence their formation 7–11 years after normal premolars [19, 20].

The patient in Case  1 was a nonsyndromic female with multiple supplemental supernumerary teeth in four quadrants of her mouth. The panoramic radiograph taken prior to orthodontic treatment revealed seven supernumeraries which could interfere in space closing during orthodontic treatment and might cause complications such as resorption of adjacent roots. Therefore it was decided to remove all supernumerary teeth to prevent potential problems in orthodontic treatment. Surgical removal of supernumeraries should be performed with great caution to avoid damaging adjacent permanent teeth, which may cause ankylosis of these teeth and consequently may create difficulty in the course of orthodontic treatment. Moreover the possibility of supernumeraries' fusion to the neighboring teeth should be kept in mind [21, 22].

In Case  2 a supernumerary premolar was found by chance in the panoramic radiograph taken for assessment of third molar teeth. Patient had undergone a course of orthodontic treatment at age 13, and there was no sign of a supernumerary tooth in the available preorthodontic panoramic radiograph. In this case it was unlikely that the supernumerary premolar caused any interference in terms of function or esthetic. It has been reported that pathological changes occur in merely 2 percent of supernumerary premolars [23]. Based on these facts it was decided to leave the supernumerary teeth and to keep the condition under periodic radiographic observation.

## 4. Conclusion

Orthodontists should be aware that late development of supernumeraries can occur any time in the course of orthodontic treatment or after its completion. Although it is not a routine practice to screen for the late development of teeth during or after orthodontic treatment, possibility of their complications should always be taken into consideration.
